# Development and Validation of a Prediction Model for Co-Occurring Moderate-to-Severe Anxiety Symptoms in First-Episode and Drug Naïve Patients With Major Depressive Disorder

**DOI:** 10.1155/da/9950256

**Published:** 2024-11-19

**Authors:** Xiao Huang, Xiang-Yang Zhang

**Affiliations:** ^1^Department of Anesthesiology, Beijing Chao-Yang Hospital, Capital Medical University, No. 8 Workers' Stadium South Road, Beijing 100020, Chaoyang Distinct, China; ^2^Department of Psychiatry, Hefei Fourth People's Hospital, Hefei, China; ^3^Department of Psychiatry, Affiliated Mental Health Center, Anhui Medical University, Hefei, China

## Abstract

**Background:** Moderate-to-severe anxiety symptoms are severe and common in patients with major depressive disorder (MDD) and have a significant impact on MDD patients and their families. The main objective of this study was to develop a risk prediction model for moderate-to-severe anxiety in MDD patients to make the detection more accurate and effective.

**Methods:** We conducted a cross-sectional survey and tested biochemical indicators in 1718 first-episode and drug naïve (FEDN) patients with MDD. Using machine learning, we developed a risk prediction model for moderate-to-severe anxiety in these FEDN patients with MDD.

**Results:** Four predictors were identified from a total of 21 variables studied by least absolute shrinkage and selection operator (LASSO) regression analysis, namely psychotic symptoms, suicide attempts, thyroid stimulating hormone (TSH), and Hamilton Depression Scale (HAMD) total score. The model built from the four predictors showed good predictive power, with an area under the receiver operating characteristic (ROC) curve of 0.903 for the training set and 0.896 for the validation set. The decision curve analysis (DCA) curve indicated that the nomogram could be applied to clinical practice if the risk thresholds were between 13% and 40%. In the external validation, the risk threshold was between 14% and 40%.

**Conclusion:** The inclusion of psychotic symptoms, suicide attempts, TSH, and HAMD in the risk nomogram may improve its utility in identifying patients with MDD at risk of moderate-to-severe anxiety. It may be helpful in clinical decision-making or for conferring with patients, especially in risk-based interventions.

## 1. Introduction

Major depressive disorder (MDD) is a highly prevalent, multisymptomatic, and debilitating disorder that is the third leading cause of disability worldwide [1]. It is characterized by physical changes such as fatigue, weight loss, and loss of appetite. The 1-year prevalence of MDD ranges from 4% to 5% in adolescents, with lifetime prevalence rates as high as 11% [2, 3], and 13.3% in the elderly [4]. Both psychosocial stress and systemic illnesses can influence the development of depression. In addition to the well-known psychological causes, MDD is associated with genetic factors, social anxiety, and even other prevalent chronic diseases.

The presence of MDD is a risk factor for a variety of comorbidities, including anxiety and metabolic disorders. The prevalence of depression and anxiety disorders has been increasing in recent years. Anxiety is characterized by uncontrollable fear and worry about a variety of events or activities, nearly one-third of people are diagnosed with depression and one-quarter with anxiety during their lifetime [5]. Anxiety has a significant impact on life satisfaction and health and leads to increased health burden [6]. Anxiety disorders and MDD share a common genetic predisposition [7]. In contrast to sadness alone, anxious depression has been recognized as a subtype of MDD and is linked to heightened immunological dysregulation, cortical thinning, and cortical limbic dysfunction [8]. Anxiety-related MDD patients often experience more severe depressive episode [9], worse clinical course [10], more comorbid illnesses [11], and functional impairments than MDD patients without anxiety. In patients with first-episode and drug naïve (FEDN) MDD, comorbid moderate-to-severe anxiety is especially significant due to its possible influence on future diagnosis and therapy. As a result, accurate early detection of moderate-to-severe anxiety is needed for improving the prognosis of MDD patients.

According to published research, prevalence of anxiety in MDD ranges from 42% to 78% [8]. Fava et al. [12] showed that about 53.2% of outpatients with MDD had clinically significant levels of anxiety. According to Yin et al. [13], patients with MDD combined with dyslipidemia had an 11.02% prevalence of severe anxiety disorders, and suicidal history, female gender, body mass index (BMI), Hamilton Depression Scale (HAMD), Positive Symptom Scale (PSS), and free thyroxine (FT4) level were risk factors for the development of severe anxiety symptoms. Chourpiliadis et al. [14] showed that high glucose and triglyceride (TG) levels and low high-density lipoprotein-cholesterol (HDL-C) levels were associated with an increased risk of depression, anxiety, and stress-related disorders. A family history of poor mental health is a predictor for anxiety and depression [15]. Other variables including gender, age, marital status, education level, stage of illness, and the structure and functioning of social networks also predict the occurrence of anxiety and depression [16, 17]. Although many studies have emphasized the prevalence and risk factors for critical levels of depression and anxiety disorders based on diagnostic criteria, study-identified risk prediction models for moderate-to-severe anxiety in patients with FEDN MDD remain limited.

Because of the high prevalence of anxiety in patients with MDD, systematic screening for moderate-to-severe anxiety should be conducted to improve early detection and prevention of comorbidities. Therefore, in the present study, data were collected on a large scale from patients with MDD who had a first-episode and were receiving no medication. In this context, clinical predictive modeling can objectively reveal the risk of moderate-to-severe anxiety in patients with MDD, thereby informing clinical decision-making. To the best of our knowledge, the present study was the first to build a machine-learning predictive algorithm based on these data, aiming to detect moderate-to-severe anxiety in patients with MDD.

## 2. Participants and Methods

### 2.1. Participants

This study was conducted in the psychiatric outpatient clinic of a general hospital in Taiyuan, Shanxi Province, China. We interviewed all outpatients and then consecutively recruited all cases that met the inclusion and exclusion criteria. The inclusion criteria were as follows: (1) between 18 and 60 years of age and Han nationality; (2) diagnosis of MDD according to the Diagnostic and Statistical Manual of Mental Disorders, Fourth Edition (DSM-IV), with the duration of this episode not longer than 24 months; (3) first-episode disease; (4) no history of antidepressant, antipsychotic, other psychotropic medications (e.g., benzodiazepines, mood stabilizers, etc.) or any medications that may affect thyroid function, blood glucose, or lipid levels; (5) ability to participate in the clinical assessment; and (6) total score of equal to or greater than 24 points on the HAMD. A total of 1796 patients were eligible for the study.

Patients with psychiatric disorders, drug or alcohol abuse, neurodegenerative and neurological disorders, pregnancy or breastfeeding, or inability to provide informed consent were excluded. Seventy-eight participants were excluded for the following reasons: (1) refusal to participate in this study (*n* = 21); (2) pregnancy or breastfeeding (*n* = 10); (3) serious physical illness (*n* = 9); (4) substance abuse (*n* = 9); (5) serious personality disorders (*n* = 15); (6) communication disorders (*n* = 5); and (7) other unknown reasons (*n* = 9). Finally, 1718 patients were included in the following statistical analysis. According to the formula, *n* = *Z*^2^*p* (1–*p*)/*d*^2^ [18] (*n* = number of samples; *Z* = 95% confidence interval [CI] equal to 1.96; *d* = 0.05 [5%], marginal error; *p* = expected prevalence, which we assumed to be 30%, based on previous studies) [19–21], a sample size of 355 patients was required, taking into account the elimination rate of patients. The sample size of this study was 1718, which was significantly larger than the required sample size, indicating that this study had sufficient validity.

The institutional review board of the First Hospital of Shanxi Medical University authorized this study (No. 2016-Y27). After a thorough briefing on the study protocol and procedures by a research psychiatrist, each participant signed a written informed consent form.

### 2.2. Clinical Measurements

We collected demographic and clinical data on each subject through a detailed questionnaire. In this study, we used the HAMD, the Hamilton Anxiety Rating Scale (HAMA), and the positive subscale of the Positive and Negative Syndrome Scale (PANSS-P) to describe depressive symptoms, anxiety symptoms, and psychotic symptoms, respectively.

The HAMA consists of 14 items measuring psychological and somatic anxiety symptoms [22]. The HAMA uses a five Likert scale with a total score of 0–56. The thresholds for the HAMA are as follows: negative anxiety symptoms (0–17); mild anxiety symptoms (18–24); moderate anxiety symptoms (25–29); and severe anxiety symptoms (≥30). In the present study, moderate-to-severe anxiety was defined as a threshold of 25 points [23]. The HAMD is a test scale containing 17 items rated on a five-point Likert scale ranging from 0 (none) to 4 (severe) [24]. In this study, patients with a HAMD score of more than 24 were identified as having depressive symptoms [25]. In the present study, we only measured the positive subscale and did not ask questions about the negative symptoms. Each item of the PANSS-P was rated on a seven-point Likert scale, with 1 indicating absence and 7 indicating high severity. Thus, the total score of the PANSS-P ranged from 7 to 49. Patients with a PANSS-P subscore above 15 were considered to have psychotic symptoms [26].

Suicide attempts were documented through face-to-face interviews. All participants were requested to answer the following question: “Have you ever attempted suicide in your past life?” Those who answered “yes” were documented as having suicide attempts. These participants were further questioned about the specific date, frequency, and mode of their suicide attempts. If necessary, their families were also interviewed or contacted for further information about the details of the suicide attempts. If the answer was uncertain, their family members, relatives, or friends were contacted.

The two psychiatrists who participated in this study were trained in the scoring of the HAMD, the HAMA, and the PANSS-P scales before the clinical assessment. After training, the interclass correlation for the two psychiatrists was between 0.82 and 0.85 for the clinical assessment of the above three scales.

### 2.3. Biomarker Measurements

Blood samples were collected in the morning after an overnight fasting. All samples were sent to the laboratory center of the hospital immediately after collection and were analyzed on the same day. The tests included serum-free triiodothyronine (FT3), FT4, thyroid stimulating hormone (TSH), anti-thyroglobulin (TgAb), thyroid peroxidases antibody (TPOAb), TGs, total cholesterol (TC), low-density lipoprotein-cholesterol (LDL-C), HDL-C, and fasting blood glucose. The nurse weighed the patient's weight, height, and blood pressure. BMI was derived by the following formula:

  $$\begin{array}{c} \text{BMI}=\text{Weight (kg)/height }{\text{(m)}}^{2}. \end{array}$$

### 2.4. Statistical Analysis

According to the Kolmogorov–Smirnov test, the continuous data in this study were not normally distributed. Therefore, we expressed continuous data as median and interquartile range (IQR; 25%–75%) and categorical values in terms of frequencies and percentages. The Whitney *U* test was used for continuous data and the *χ*^2^ test for categorical data.

We randomly divided the data into training and validation sets on a 70%–30% scale and subdivided them at the patient level. We compared demographic and clinical data between patients with and without moderate-to-severe anxiety, or between the validation and confirmation sets, respectively. We used both training and validation data for model fitting and hyperparameter tuning. Then, we used a regularized regression model fitting algorithm based on the least absolute shrinkage and selection operator (LASSO). Yang et al. [27] performed LASSO and this database to develop a good predictive model about suicide risk in patients with MDD. LASSO generates a regression-based model that stabilizes the regression coefficients by variable selection. To obtain a subset of predictors, the LASSO regression analysis minimizes the prediction error of the quantitative response variables by imposing constraints on the model parameters and shrinking the regression coefficients of some variables to zero. Covariates with zero regression coefficients were excluded from the model, whereas covariates with nonzero regression coefficients had the most significant correlations with the response variables. We set moderate-to-severe anxiety as a “binomial,” that is, a dependent variable containing the presence or absence of moderate-to-severe anxiety. The included variables were pooled and normalized according to the 2log-likelihood type measure and binomial moderate-to-severe anxiety by k-fold (10-fold in this case) cross-validation using LASSO regression analyses to select the optimal *λ* values. Lambda.1se provides a well-performing model with a very small number of independent variables. Therefore, we analyzed the training set data using the LASSO method to select the best determinants of current risk factors including age, age at onset, duration of illness, marital status, education, BMI, HAMD, PANSS-P, suicide attempts, psychotic symptoms, TSH, TSH levels, A-TG, A-TPO, blood glucose, TC, HDL-C, TG, LDL-C, systolic blood pressure (SBP), and diastolic blood pressure (DBP) (*p*  < 0.1 in univariate analysis). Next, we used multivariate logistic regression analyses to build a predictive model by introducing the features selected in the LASSO regression model. By listing all selected features and analyzing statistical significance levels, we constructed a predictive model for moderate-to-severe anxiety risk using statistically significant predictors. In our study, all selected features were significant and were used to build nomogram predictive models.

We assessed the predictive performance of the training and validation data using the following three indicators: (1) receiver operating characteristic (ROC) curve, which provides a composite indicator of risk stratification for sensitivity and specificity tradeoffs. It represents an overall metric of potential clinical utility, taking into account both sensitivity and positive predictive value; (2) calibration (slope and large range), which provides an overall metric of risk-probability accuracy using the Hosmer–Lemeshow (HL) test; and (3) decision curve analysis (DCA), which determines the clinical utility of the nomogram based on the net benefit of the probability of different thresholds in the cohort. We analyzed these metrics at the patient level, using bootstrapping and resampling to calculate 95% CIs for differences between them. We calculated regression coefficients for LASSO to better understand the main predictor variables of the model.

*p* values were two-sided with a significance level of less than 0.05. The independence of the predictor variables (lack of collinearity) was assessed using a variance inflation factor (VIF) with a cutoff value of 2. Bonferroni correction was applied for multiple tests. Data were analyzed using the Statistical Package for the Social Sciences (SPSSs Version 26) and R 4.3.3.

## 3. Results

### 3.1. Characteristics of the Study Population

**Figure 1:**
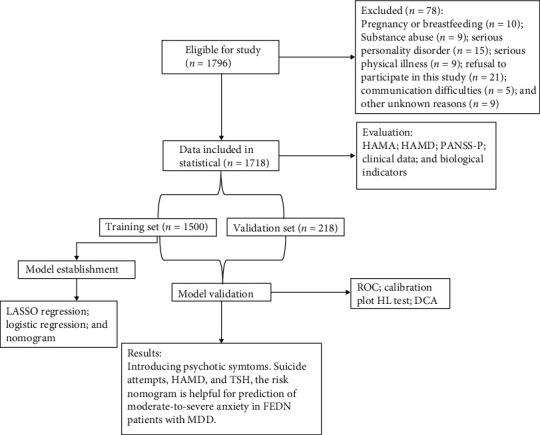
Flow diagram of study design. DCA, decision curve analysis; FEDN, first-episode and drug naïve; HAMA, Hamilton Anxiety Scale; HAMD, Hamilton Depression Scale; HL, Hosmer–Lemeshow; LASSO, least absolute shrinkage and selection operator; MDD, major depressive disorder; PANSS, Positive and Negative Syndrome Scale; ROC, receiver operating characteristic.

The enrollment flowchart is shown in Figure 1. Out of 1718 patients, 338 (19.7%) had no anxiety, 1162 (67.6%) had mild anxiety, 193 (11.2%) had moderate anxiety, and 25 (1.5%) had severe anxiety. Thus, the proportion of patients with moderate-to-severe anxiety was 12.7%. A total of 218 patients were included in the moderate-to-severe anxiety group and 1500 patients were included in the no moderate-to-severe anxiety group. These patients were randomly assigned to either the training or validation set in a 7 : 3, that is, 1203 and 515 patients were assigned to the training and validation sets, respectively.  Table 1 summarizes the demographic and clinical characteristics of patients in the two groups. All characteristics were comparable in both the training and validation sets.

Table 1 shows that 30.3% of patients with moderate-to-severe anxiety and 34.8% of patients without moderate-to-severe anxiety were male. Among patients with moderate-to-severe anxiety disorders, 78% had abnormal thyrotropin hormones (thyrotropin >4.2 mIU/L), 76.6% were married, 54.1% had suicide attempts, and 56.4% had psychotic symptoms. In addition, there was a significant difference between patients with and without moderate-to-severe anxiety in terms of age, age at onset, duration of illness, HAMD, HAMA, PANSS-P, fasting glucose, BMI, HDL-C, LDL-C, TG, TC, TSH levels, abnormal TSH, TgAb, TPOAb, SBP, and DBP (all of which passed Bonferroni correction, except for age at onset, BMI and TG).

### 3.2. Establishing the Model

**Figure 2:**
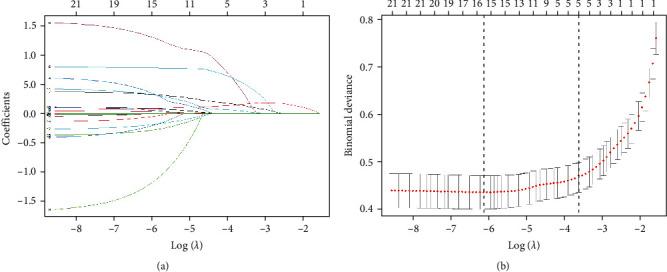
Variable selection by the LASSO binary logistic regression model. A coefficient profile plot was constructed against the log (*λ*) sequence. (a) Five variables with nonzero coefficients were selected by deriving the optimal *λ*. (b) Following verification of the optimal parameter (*λ*) in the LASSO model (dotted line on the left side of (b)), the partial likelihood deviance (binomial deviance) curve versus log (*λ*) is plotted and drawn based on 1 standard error criteria (dotted line on the right side of (b)). LASSO, least absolute shrinkage and selection operator.

**Figure 3:**
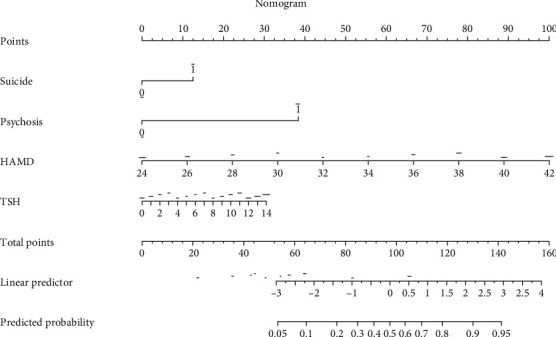
A risk factors of suicide attempts, psychotic symptoms, HAMD, and TSH for the nomogram prediction model. HAMD, Hamilton Depression Scale; TSH, thyroid stimulating hormone.

LASSO regression analysis was performed to select possible predictor variables from those listed in Table 1, including age, age at onset, education, duration of disease, HAMD and PANSS-P, fasting blood glucose, BMI, HDL-C, LDL-C, TG, TC, TSH, TSH levels, TgAb, TPOAb, SBP, and DBP. A predictive model was developed by multivariate logistic regression. The coefficients of these five variables (suicide attempts, psychotic symptoms, PANSS-P, HAMD, and TSH) were not zero in the LASSO regression model (Figure 2).  Table 2 shows the results of the logistic regression analyses of these five parameters using the entry method. Four of the predictors, including psychotic symptoms, suicide attempts, HAMD, and TSH, were statistically significantly different. They were independent of each other and were included in the predictive model to form a nomogram for moderate-to-severe anxiety (Figure 3).

The highest risk score was HAMD, with a score of 100 and HAMD was 42. The second risk factor was psychotic symptoms. Combined with psychotic symptoms, the risk score was ~38. TSH and suicide attempts were the third and fourth factors contributing to the risk score for moderate-to-severe anxiety in patients with MDD. We derived scores for each prediction based on the nomogram model. The sum of these scores corresponded to the predicted probability that an MDD patient with MDD had moderate-to-severe anxiety. For example, a patient with MDD who had a TSH level of 9.2, a HAMD score of 34, psychotic symptoms, and suicide attempts had an estimated 86% probability of having moderate-to-severe anxiety (Figure 3).

### 3.3. Validation of the Predictive Model

**Figure 4:**
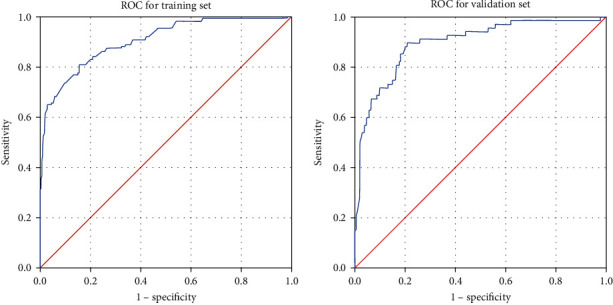
ROC validation of the moderate-to-severe anxiety nomogram prediction. The *y*-axis indicates the true positive rate of risk prediction and the *x*-axis indicates the false positive rate of risk prediction. The thick blue line indicates the performance of the nomogram in the training set and validation set. ROC, receiver operating characteristic.

The discriminative power of the predictive model was assessed by ROC. For the predictive model, the area under the curve (AUC) of the nomogram was 0.903 (95% CI: 0.875–0.931) for the training set and 0.896 (95% CI: 0.852–0.940) for the validation set, which showed good performance (Figure 4).

**Figure 5:**
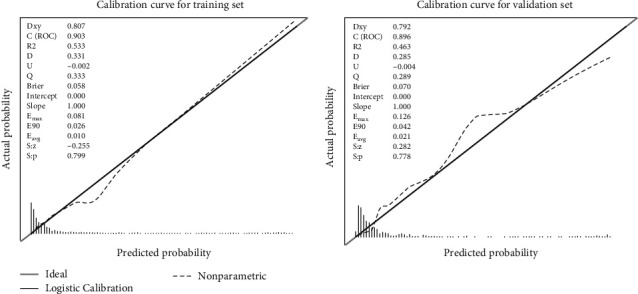
Calibration curves of the predictive moderate-to-severe anxiety risk nomogram. The *y*-axis represents actual diagnosed cases of moderate-to-severe anxiety, the *x*-axis represents the predicted risk of moderate-to-severe anxiety. The diagonal gray line represents a perfect prediction by an ideal model, and the dotted line represents the performance of the training set and validation set, with the results showing that a closer fit to the ideal line represents a better prediction.

**Figure 6:**
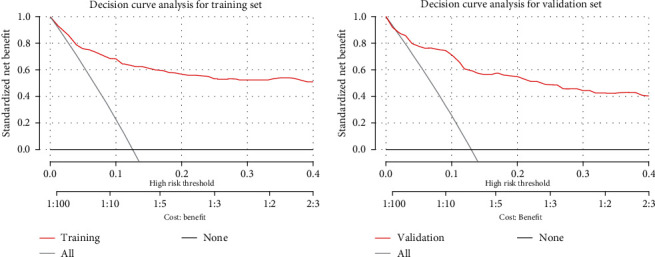
Decision curve analysis for the moderate-to-severe anxiety risk nomogram. The *y*-axis measures the net benefit. The gray line represents the assumption that all patients have moderate-to-severe anxiety, the black line represents the assumption that all patients have no moderate-to-severe anxiety, and the red line represents the risk nomogram.

A calibration plot and the HL test were carried out to calibrate the predictive model. The calibration curves showed that both the predictive model and the validation set fitted very well. The HL test indicated that the predicted probabilities were highly consistent with the actual probabilities (training set, *χ*^2^ = 13.794, *p* = 0.087; validation set, *χ*^2^ = 9.628, *p* = 0.292) (Figure 5). DCA showed that the threshold probabilities of the predictive model in the training and validation sets were 13%–40% and 14%–40%, respectively (Figure 6). The Brier score was 0.058 for the predictive model and 0.07 for the validation model.

## 4. Discussion

We developed a model consisting of four variables, namely psychotic symptoms, suicide attempts, HAMD, and TSH, to predict moderate-to-severe anxiety in patients with MDD based on the predictive model.

This study has several advantages over previous studies that identified risk factors for moderate-to-severe anxiety in patients with MDD. More than 20 clinical indicators for analysis, including demographic characteristics, clinical symptoms, and biochemical indicators were used in the present study. However, previous studies have not included such a wide range of variables as our study. For example, Remmerswaal et al. [28] found that the most important risk factors for anxiety and depression were the number of psychiatric disorders at baseline, neuroticism, childhood maltreatment, parental psychopathology, and alcohol use, with AUCs of 0.68 and 0.75 for the chronic and overall course of the index disorders, respectively. A multicentre cohort study of Hispanic enrollees found childhood/adolescent emotional abuse or neglect, bipolar spectrum disorder, suicidal ideation, and depression and anxiety symptoms were the strongest predictors of first-episode depression and anxiety, with an AUC of 0.76 [29]. We used a novel statistical method to identify risk factors for moderate-to-severe anxiety. This approach is simple and efficient in modeling, with AUC for both the training and validation sets close to 9. The LASSO procedure used contraction properties to address the problem of treating different confounders as variables and the presence of multicollinearity and to provide a more stable selection of variables. We used LASSO regression for variable screening to select the cleanest model with the best predictor variables and then analyzed the LASSO regression results in a logistic regression analysis. Specifically, we randomly divided the study population into a training set and a validation set in a 7 : 3 for external validation. We generated two sets of ROC curves, calibration curves, and DCA curves to verify the accuracy and stability of the model.

The nomograms in this study were more in favor of preclinical findings and showed an association between psychotic symptoms and anxiety. First, our study found that MDD patients with moderate-to-severe anxiety had higher PANSS-P scores. A previous study also showed that psychotic-like experiences were positively associated with anxiety [30]. Psychotic-like experiences have been found to increase the risk of several psychiatric disorders such as depression and anxiety [31]. Several factors may contribute to the relationship between anxiety, depression, and psychotic symptoms. Positive and negative psychotic symptoms may share common, generalized psychopathological factors with anxiety. Anxiety decreases motivation to participate in social life and daily activities, which can lead to psychosis-like experiences [31]. Cognitive distortions also mediate the relationship among anxiety, depression, and psychotic symptoms [32]. Furthermore, psychosis can lead to sudden and unpredictable mood swings, which can increase the risk of anxiety [33].

Second, our research revealed that among patients with MDD, suicide attempts were associated with a higher likelihood of experiencing moderate-to-severe anxiety. The study also found that a significant percentage of the patients had suicide attempts in the past, with a high incidence of 20.1%. The following could be the cause of the suicidality of participants who made an attempt during their first depressive episode: First, among the individuals in this study, the duration of MDD was not longer than 24 months. We only included patients who were not receiving psychiatric medication treatment. So these patients experiencing MDD in the past year while not taking any medications were likely to develop suicidal tendencies. Second, a strong correlation has been demonstrated between a higher severity of depression and additionally suicidality [34, 35]. Of the patients with FEDN MDD during the COVID-19 pandemic, Cui et al. [36] discovered that 14.4% had made an attempt at suicide. According to a study by Tian et al. [37], 47.7% of FEDN MDD patients reported having suicidal thoughts in the previous week. Using our database, Ye et al. [38] found that the rate of suicide attempts was 19.5% among young MDD patients. A meta-analysis revealed that 15.80% and 32.30% of MDD outpatients and inpatients, respectively, had attempted suicide [39]. Additionally, numerous studies have also shown that anxiety increases the likelihood of suicide in MDD patients. As an illustration, a 5-year longitudinal study showed significant differences in anxiety levels between depressed patients with and without suicide attempts [40]. Moller et al. [41] found that among treatment-seeking adolescents with MDD, symptoms of depression and anxiety independently predicted higher suicidal ideation. Anxiety itself, as well as coexisting depression, is known to be causally related to suicide risk [42]. The coexistence of substance abuse and suicide attempts also increased anxiety rates. Psychological autopsy studies have found that the majority of suicidal individuals exhibit at least subthreshold psychiatric symptoms, suggesting that suicide risk may be a warning sign of anxiety [43]. Anxiety disorders are more common in patients who suffer from depression and suicide, and they are also exacerbated by each other.

Third, our findings showed that HAMD was associated with moderate-to-severe anxiety in patients with MDD. Previous studies have also shown that patients with anxious depression are more likely to have symptoms related to generalized anxiety disorder, obsessive–compulsive disorder, panic disorder, and somatoform disorder [44]. It is well-known that there is a strong correlation between anxiety and depression in the general population. For example, Lamers et al. [45] found that the severity and duration of depressive symptoms were usually higher in patients with MDD with comorbid anxiety. A common explanation is that anxiety and depression require similar changes in neural circuitry, and that serotonin reuptake inhibitors (e.g., sertraline), which are used in the treatment of depression, may reduce anxiety symptoms. In addition, some lifestyle changes (e.g., sleep disorders) also explain the role of depressive symptoms in anxiety [46]. This suggests that we should pay more attention to the association between depression and anxiety, investigate the associated risk factors, and promote the development of preventive and intervention measures. Our findings further confirm that MDD patients with comorbid anxiety suffered from severe depression, suggesting greater difficulties and challenges in treating this more complex manifestation of depression.

Fourth, we showed that elevated TSH was a predictor for moderate-to-severe anxiety in patients with MDD, which is consistent with previous studies [47]. In anxiety disorders including panic disorder and social anxiety disorder, the more severe the anxiety symptoms, the higher the serum TSH levels [48]. The following reasons may explain the mechanism by which TSH increases the risk of anxiety. TSH indicates the presence of thyroid disease or an increased risk of thyroid disease. Anxiety disorders (including panic disorder and social anxiety disorder) and depression are known to be associated with abnormalities in thyroid function, such as elevated serum TSH [49]. The etiology of anxiety disorders is complex and includes biological, clinical, psychological, and environmental factors. In addition, several studies have shown extensive structural changes in white matter (WM) and gray matter (GM) in patients with thyroid dysfunction, affecting mood, cognitive regulation, and visual functional areas [50, 51]. Furthermore, hypothalamic–pituitary–adrenal (HPA) axis dysfunction is one of the hypotheses explaining the pathogenesis of MDD [52]. All these suggest a high degree of pathophysiological overlap between these two conditions.

### 4.1. Limitations

There are several limitations to this study. First, because of the cross-sectional design of this study, it is not entirely clear whether there is a causal relationship between anxiety and these risk factors. In addition, this cross-sectional study only identified these patients as having psychotic symptoms but did not differentiate whether all of them had psychotic depression or whether some of the patients' psychotic symptoms were caused by other mental disorders. Second, this study could not rule out many unidentified confounding factors associated with anxiety, including genetic factors, psychosocial factors, and life events. Third, levels of thyroid hormones and metabolic indicators may change over time. However, dynamic assessment was limited in our study. Fourth, the participants in this study were from the psychiatric outpatient clinic of a general hospital. Therefore, the results of this study cannot be generalized to the overall population. Fifth, the HAMA and PANSS scales cannot be used to accurately diagnose anxiety and psychiatric disorders, but only to quantify symptoms. In addition, in the present study, we only used the HAMA scale to determine whether the patients suffered from anxiety symptoms; therefore, comorbidities with other DSM-IV mental disorders, such as anxiety disorders, were not available in the present study, which should be taken into account in future studies. Finally, in this study, all participants were patients with FEDN MDD with a HAMD total score higher than 24. Therefore, our results cannot be generalized to all Chinese patients with MDD.

## 5. Conclusion

Our results showed that the four indicators validated by the nomogram in this study, including psychotic symptoms, suicide attempts, HAMD, and TSH, were highly significant in differentiating the risk of moderate-to-severe anxiety in patients with MDD. Our prediction model covers both clinical scales and laboratory tests, and these indicators are involved in psychiatric patients generally, making it more accurate to predict moderate-to-severe anxiety and facilitating clinical decision-making, and crisis intervention for MDD patients timely when compared to using a questionnaire or conducting a clinical interview. To increase the predictive accuracy of the model, additional testing and updates are required when more clinical data become available.

## Figures and Tables

**Table 1:** Characteristics of the 1718 MDD enrolled in the study according to with or without moderate-to-severe anxiety and randomization to the training set and validation set.

Items	Patients without moderate-to-severe anxiety (*n* = 1500) *M* (Q25–Q75) or *N* (%)	Patients with moderate-to-severe anxiety (*n* = 218) *M* (Q25–Q75) or *N* (%)	*χ* ^2^/*F*/*Z*	*p* value	Training set (*n* = 1203) *M* (Q25–Q75) or *N* (%)	Validation set (*n* = 515) *M* (Q25–Q75) or *N* (%)	*χ* ^2^/*F*/*Z*	*p* value
Age (years)	33.5 (23, 45)	36 (26, 48)	−2.308	<0.001	34 (23, 46)	34 (24, 45)	−0.204	0.839
Illness duration (months)	5 (3, 8)	6 (4, 9)	−4.164	<0.001	5 (3, 8)	5 (3, 8)	−0.537	0.591
Age onset (years)	33 (23, 45)	35.5 (26, 47.3)	−2.269	0.023	33 (23, 45)	34 (24, 45)	−0.223	0.823
Gender (male)	522 (34.8)	66 (30.3)	1.731	0.188	419 (34.8)	169 (32.8)	0.65	0.42
Education level, *n* (%)	—	—	7.389	0.06	—	—	2.872	0.412
Middle school	345 (23)	68 (3.1)	—	—	281 (23.4)	132 (25.6)	—	—
High school	674 (44.9)	86 (39.4)	—	—	534 (44.4)	226 (43.9)	—	—
College	398 (26.5)	51 (23.4)	—	—	325 (27)	124 (24.1)	—	—
Graduate	83 (5.5)	13 (6)	—	—	63 (5.2)	33 (6.4)	—	—
Married	1049 (69.9)	167 (76.6)	4.097	0.043	850 (70.7)	366 (71.1)	0.029	0.864
BMI (kg/m^2^)	24.2 (23.2, 25.6)	24.59 (23.6, 26.1)	−3.099	0.002	24.3 (23.2, 25.6)	24.2 (23.2, 25.5)	−1.301	0.193
HAMD	30 (28, 32)	33 (32, 36)	−16.162	<0.001	30 (28, 32)	30 (28, 32)	−0.068	0.946
HAMA	20 (18, 22)	27 (26, 28)	−23.99	<0.001	21 (18, 23)	21 (18, 23)	−0.073	0.942
PANSS-P	7 (7, 7)	19 (8, 21)	−21.713	<0.001	7 (7, 8)	7 (7, 7)	−0.1	0.92
Suicide attempts	228 (15.2)	118 (54.1)	179.339	<0.001	237 (19.7)	109 (21.2)	0.481	0.488
With psychotic symptoms	48 (3.2)	123 (56.4)	601.544	<0.001	121 (10.1)	50 (9.7)	0.049	0.825
TSH (mIU/L)	4.7 (2.9, 6.3)	7.25 (5.2, 9.4)	−12.241	<0.001	4.9 (3.2, 6.7)	4.9 (3, 6.6)	−0.282	0.778
TSH level (>4.2 mIU/L)	874 (58.3)	170 (78)	31.032	<0.001	732 (60.8)	312 (60.6)	0.011	0.918
TgAb (IU/L)	21.2 (14.1, 37)	23.7 (16.4, 119.1)	−4.759	<0.001	21.8 (14.2, 46.9)	21 (15, 37.8)	−0.097	0.922
TPOAb (IU/L)	16.8 (12.2, 32.5)	24.3 (13.2, 122.6)	−5.028	<0.001	17 (12.3, 34.1)	18.7 (12.5, 35.3)	−0.957	0.339
FT3 (pmol/L)	4.9 (4.4, 5.4)	4.9 (4.4, 5.4)	−0.166	0.868	4.9 (4.4, 5.4)	4.9 (4.4, 5.4)	−0.796	0.426
FT4 (pmol/L)	16.5 (14.3, 18.7)	16.7 (14.7, 18.9)	−1.625	0.104	16.6 (14.5, 18.7)	16.4 (14.3, 18.8)	−0.523	0.601
Fasting glucose (mmol/L)	5.3 (4.9, 5.8)	5.5 (5.1, 6.1)	−4.731	<0.001	5.4 (5, 5.8)	5.3 (4.9, 5.9)	−0.089	0.929
TC (mmol/L)	5.1 (4.4, 5.9)	5.6 (4.8, 6.5)	−6.398	<0.001	5.2 (4.4, 6)	5.2 (4.5, 6)	−0.86	0.39
HDL-C (mmol/L)	1.2 (1, 1.4)	1.1 (0.9, 1.3)	−4.526	<0.001	1.2 (1, 1.4)	1.2 (1, 1.4)	−0.707	0.48
TG (mmol/L)	1.9 (1.4, 2.8)	2.2 (1.4, 3)	−2.419	0.016	2 (1.4, 2.8)	1.9 (1.4, 2.8)	−1.512	0.131
LDL-C (mmol/L)	2.9 (2.3, 3.5)	3.2 (2.7, 3.7)	−4.261	<0.001	3 (2.4, 3.6)	2.9 (2.4, 3.5)	−0.191	0.848
SBP (mmHg)	120 (111, 126)	125 (118, 132)	−7.288	<0.001	120 (112, 126)	120 (112, 128)	−0.577	0.564
DBP (mmHg)	75 (70, 80)	78 (74, 82.3)	−5.396	<0.001	75 (70, 80)	76 (72, 80)	−1.599	0.11

*Note:* We used the Mann–Whitney *U* test for non-normal variables and the *χ*^2^ test for categorical variables.

Abbreviations: BMI, body mass index; DBP, diastolic blood pressure; FT3, free triiodothyronine; FT4, free thyroxine; HAMD, Hamilton Depression Scale; HDL-C, high-density lipoprotein-cholesterol; LDL-C, low-density lipoprotein-cholesterol; PANSS-P, Positive Subscale of the Positive and Negative Symptom Scale; SBP, systolic blood pressure; TC, total cholesterol; TG, triglyceride; TgAb, anti-thyroglobulin; TPOAb, thyroid peroxidases antibody; TSH, thyroid stimulating hormone.

**Table 2:** Logistic regression analysis of the predictors for the risk of moderate-to-severe anxiety in patients with MDD.

95% confidence interval for EXP (*B*)							
Variables	*B*	Standard error	Wald	*p* value	OR	Lower	Upper
Psychotic symptoms	1.625	0.771	4.441	0.035	5.079	1.12	23.021
Suicide attempts	0.915	0.208	19.278	<0.001	2.496	1.659	3.756
TSH	0.131	0.041	10.039	0.002	1.14	1.051	1.236
HAMD	0.287	0.05	32.406	<0.001	1.332	1.207	1.47
PANSS-positive	0.079	0.062	1.611	0.204	1.082	0.958	1.223

Abbreviations: HAMD, Hamilton Depression Scale; OR, odds ratio; PANSS, Positive and Negative Syndrome Scale; TSH, thyroid stimulating hormone.

## Data Availability

The data that support the findings of this study are available upon request from the corresponding author. The data are not publicly available due to privacy or ethical restrictions.
